# Polymer Capsules with Volatile Organic Compounds as Reference Materials for Controlled Emission

**DOI:** 10.1021/acsami.4c12826

**Published:** 2024-12-04

**Authors:** Anna Musyanovych, Christoph Grimmer, Ali Enis Sadak, Lorenz Heßling, Malin Lüdicke, Mine Bilsel, Wolfgang Horn, Matthias Richter

**Affiliations:** † Fraunhofer IMM, Carl Zeiss Str. 18-20, 55129 Mainz, Germany; ‡ Bundesanstalt für Materialforschung und -prüfung (BAM), Unter den Eichen 87, 12205 Berlin, Germany; § TUBITAK UME, Chemistry Group Laboratories, Gebze, 41470 Kocaeli, Turkiye

**Keywords:** polymer microcapsules, membrane emulsification, polyaddition, volatile organic compound (VOC), emission testing

## Abstract

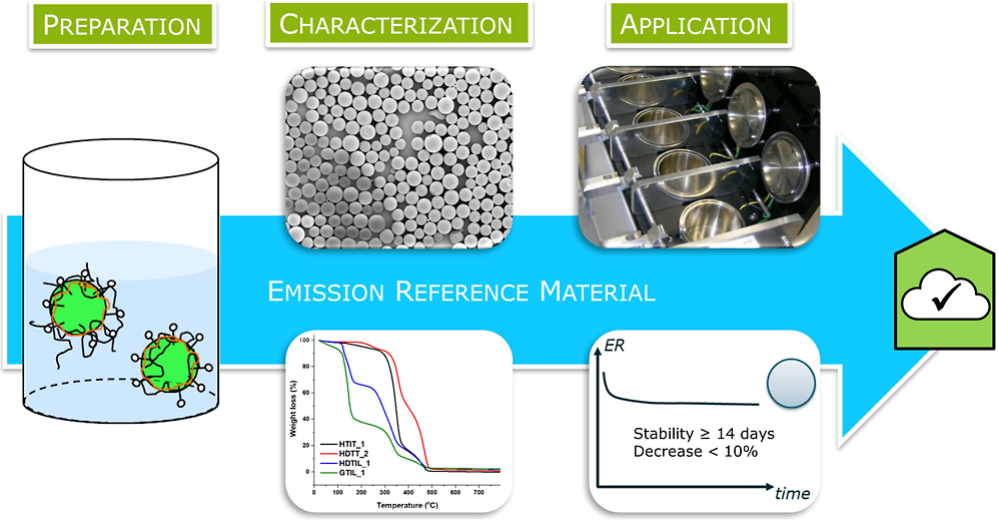

Encapsulation of volatile organic compounds (VOCs) that could evaporate at a defined rate is of immense interest for application in emission reference materials (ERMs). Polyurethane/polyurea microcapsules with various VOC active ingredients (limonene, pinene, and toluene) were successfully produced by interfacial polymerization with Shirasu porous glass membrane emulsification in a size range between 10 and 50 μm. The effect of surfactant, VOC, monomer(s) type, and ratio has a great effect on the formulation process and morphology of capsules. The type of VOC played a significant role in the encapsulation efficiency. Due to the difference in vapor pressure and VOC/water interfacial tension, the formulation for encapsulation was optimized for each individual VOC. Furthermore, to achieve effective stability of the large droplets/capsules, a combination of ionic and nonionic surfactants was used. Optical and scanning electron microscopy, Fourier transform infrared spectroscopy (FTIR), and thermogravimetric analysis (TGA), were used to characterize the optimized microcapsules. The results showed that the obtained microcapsules exhibited a spherical shape and core–shell morphology and featured characteristic urethane-urea bonds. The amount of encapsulated VOC ranges between 54 and 7 wt %. The emission tests were performed with the help of the emission test chamber procedure (EN 16516). The limonene-loaded polyurethane/polyurea microcapsules show a change in emission rate of less than 10% within 14 days and can be considered as a potential candidate for use as an ERM.

## 1 Introduction

The indoor environment has a significant influence on human health and a person’s perception of well-being. Volatile organic compounds (VOCs) emitted from construction products and other products used indoors, such as furniture, electronic devices, household products, etc., constitute a significant source of indoor pollution.^1,2^ Under certain environmental and occupational conditions, they might result in sensory irritation and in health complaints often referred to as the “sick building syndrome”.^3−7^ Emission concentrations can become further elevated in new or refurbished buildings,^8^ where the rate of air exchange with fresh ambient air may be limited due to improved energy-saving aspects.^9^ A healthy indoor environment can be achieved by controlling the sources and eliminating or limiting the release of harmful substances into the air. One way is to use materials proven to be low emitting. Meanwhile, a worldwide network of professional, commercial, and noncommercial laboratories performing emission tests for the evaluation of products for interior use has been established. For this purpose, comparability of the test results must be ensured. A laboratory’s proficiency can be proven by internal and external validation measures that both include the application of suitable reference materials. The emission test chamber procedure according to ISO 16000-9^10^ and EN 16516^11^ comprises several steps from sample preparation to sampling of test chamber air and chromatographic analysis. Quality assurance and quality control (QA/QC) must, therefore, be ensured. For this purpose, well-characterized emission reference materials (ERMs) are desirable that release a known amount of selected VOC when they are put into an emission test chamber. Suitable ERMs that emit a known amount of a compound are currently unavailable, although some promising approaches can be found in the literature. Nohr et al.^12^ and Horn et al.^13^ developed with a multi-VOC ERM based on a water-based lacquer to which a mixture of VOCs was added. After portioning into Petri dishes, the lacquer was cured and could be sent to the user. However, production reproducibility was shown only within the batch of the lacquer material, and the emission profile decreased rapidly after its application. Furthermore, colligative effects of the added VOCs in terms of miscibility, volatilization, or chemical reactions during curing were reported.^12^ To fulfill all QA/QC requirements of test standards dealing with emission test chambers, such as the determination of the recovery, sources are necessary that release the target VOC constantly over time. Wei et al.^14^ developed a tool which they called liquid-inner tube diffusion-film-emission (LIFE). It consists of a PTFE vial capped with a membrane and containing the compound of interest (toluene). Salthammer et al.^15^ published another approach of a permeation-controlled formaldehyde reference source. However, these strategies have not been further developed for use as a multi-VOC releasing source. To the best of our knowledge, no ERM is commercially available yet.

In this research, we synthesized polymer microcapsules loaded with different VOCs and investigated their emission properties. Microcapsules consisting of a solid shell and liquid core can be used to protect the encapsulated materials from harsh environmental conditions and to control their release rate and release mechanism. Among all shell materials, polyurethane/polyurea microcapsules possess high chemical, mechanical, and thermal stability. They are diversely applied in construction, energy storage, cosmetics, healthcare, consumer products, and agriculture.^16−25^ The microencapsulation of limonene with polyurethane–urea and its application on cotton fabrics was described by Yilmaz et al.^26^ These kinds of microcapsules are typically produced through interfacial polymerization, a process where a reaction between isocyanates and diols or diamines occurs at the oil–water interface. The reaction occurs very quickly and can be used for encapsulation of many hydrophobic liquids. Therefore, this method was selected to prepare VOC-loaded microcapsules in the present study.

The Shirasu porous glass (SPG) membrane emulsification was selected to produce monodisperse VOC-loaded droplets/microcapsules, due to the properties of microcapsules, such as release profile, permeability, and stability over time, often depend on particle size. SPG membrane emulsification is a very promising technique used for producing emulsions and particles with precise control over size and distribution.^27−29^ It was introduced by Nakashima et al.^30^ to prepare uniform-sized emulsion and later developed by Omi and Ma et al.^31−33^ to prepare monodisperse microspheres by polymerizing uniform monomer droplets. In SPG membrane emulsification, the dispersed phase (typically oil) is forced through the pores of the SPG membrane into the continuous phase (typically water), creating droplets. The size of these droplets is primarily determined by the pore size of the membrane and the operating conditions, such as pressure and flow rate. Moreover, SPG membrane emulsification requires less energy compared to conventional emulsification methods, making it more efficient and cost-effective.

In the current work, we investigated the factors influencing the formation of stable and monodisperse polyurethane/polyurea microcapsules loaded with three different VOCs (limonene, toluene, and pinene). Two SPG membranes with different pore sizes were applied, and suitable conditions were found for the generation of microcapsules possessing an effective emission of the encapsulated VOCs.

## 2 Experimental Section

### 2.1 Chemicals and Reagents

All chemicals used within the project were of analytical grade and used as received without further purification. d-limonene (96%, Thermo Scientific), α-pinene (98%, Aldrich), toluene (99.9%, VWR Chemicals), glycerin (99%, abcr GmbH and Co., KG), 1,6-hexanediol (99%, Sigma-Aldrich), 1,6-hexanediamine (HD, Merck), isophorone diisocyanate (IPDI, 98%, Acros Organics), tolylene 2,4-diisocyanate (TDI, 95%, Sigma-Aldrich), TUBASSISTFIX 157W (Tub, CHT R. BEITLICH GmbH), a solvent-free commercial product based on hexamethylene diisocyanate oligomers, and dibutyltin dilaurate (DBTL, 95%, Sigma-Aldrich). Surfactants: poly(vinyl alcohol) (PVA, Mw 31,000 g/mol, ≥99.5%, Sigma-Aldrich) and sodium dodecyl sulfate (SDS, 98%, Acros Organics). Ultrapure water (Thermo Scientific) was used throughout the experimental runs.

### 2.2 Preparation of Capsules

**Figure 1:**
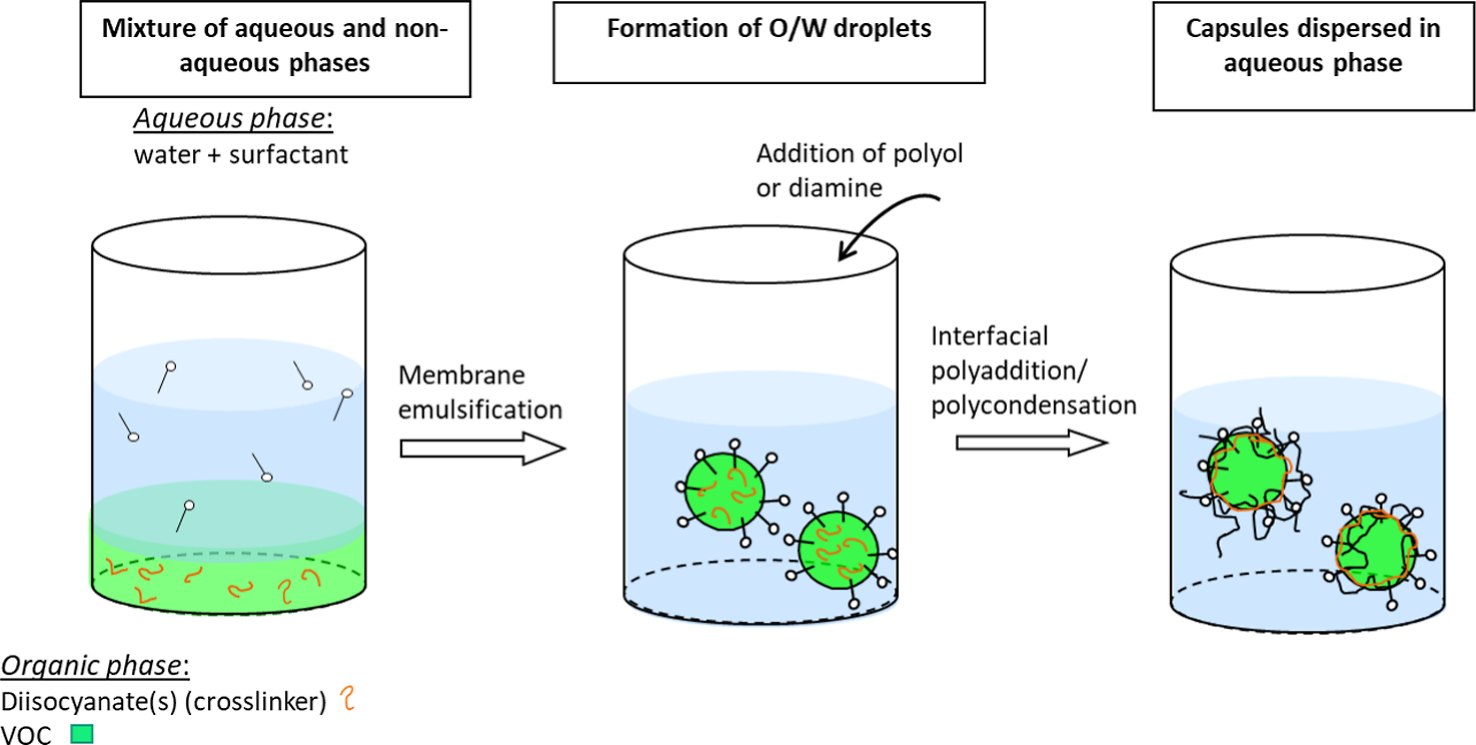
Scheme of the synthetic process for VOC-microcapsule preparation through interfacial polymerization.

The VOC-loaded capsules were prepared by a polyaddition/polycondensation reaction performed at the oil-in-water (O/W) emulsion droplet’s interface. The droplets were generated using a SPG membrane emulsification module (External Pressure Micro Kit (MG-20), SPG Technology Co., Ltd., Japan). For the formation of capsules with an average size of 15 and 50 μm, the SPG membranes were used with the pore size of 5.1 and 9.9, respectively. The dispersed phase, consisting of VOC, DBTL (in some runs), and isocyanate(s), was pressed into the continuous phase through the SPG membrane using pressurized nitrogen gas. The continuous phase consists of surfactant(s) in aqueous solution. After the whole organic phase was dispersed, the emulsion was placed in a heated oil bath at 60 or 45 °C (when 9.9 μm SPG membrane was used), and the hydrophilic monomer (glycerin, hexanediol, or hexamethylenediamine) was added. The polyaddition reaction proceeded overnight with magnetic stirring at 300 rpm. The synthetic process was illustrated for VOC-microcapsule preparation through interfacial polymerization in Figure 1, and the details are summarized in Table 1 regarding the compositions of the two phases.

**Table 1:** Chemical Composition and Average Size of Capsules Loaded with VOC^a^

sample name	VOC, mL	cross-linker/catalyst	hydrophilic monomer	surfactant	size, μm
with limonene					
SPG membrane 5.1 μm					
GTIL_1	4.7	IPDI 0.92 g + Tub 0.2 g	glycerin, 0.1 g	40 mL PVA, 1 wt %	16 ± 4
HDTIL_1			HD, 0.5 g		21 ± 10
GTIL_110Ao	2.1	IPDI 0.6 g + Tub 0.15 + DBTL 6 μL	glycerin, 0.1 g		43 ± 8
HDTIL_109Bu			HD, 0.15 g		24 ± 6
SPG membrane 9.9 μm					
HDTIL_120A	4.3	IPDI 1.2 g + Tub 0.3 g + DBTL 12 μL	HD, 0.5 g	120 mL PVA, 1 wt % + SDS 0.2 wt %	58 ± 6
HDTTL_133A	2.0	TDI 0.125 g + Tub 0.125 g	HD, 0.16 g	60 mL PVA, 1 wt % + SDS 0.2 wt %	37 ± 7
HDTTL_143	4.0	TDI 0.34 g + Tub 0.17 g	HD, 0.33 g	120 mL PVA, 1 wt % + SDS, 0.2 wt %	60 ± 10
with toluene					
SPG membrane 5.1 μm					
HTIT_1	4.7	IPDI, 0.92 g + Tub, 0.21 g + DBTL, 7.5 μL	hexanediol, 0.1 g	40 mL PVA, 1 wt %	44 ± 19
GTIT_1			glycerin, 0.1 g		37 ± 25
HDTT_2		Tub, 0.8 g	HD, 0.5 g	40 mL SDS, 0.23 wt %	43 ± 11
with pinene					
SPG membrane 5.1 μm					
GTIP_2	4.7	IPDI, 0.92 g + Tub, 0.2 g	glycerin, 0.1 g	40 mL PVA, 1 wt %	19 ± 5
HDTIP_1			HD, 0.5 g		23 ± 7
HTIP_1			hexanediol, 0.1 g		20 ± 10

a The sample names are composed as follows: the first letter gives the type of hydrophilic monomer (G—glycerine, HD—hexanediamine, and H—hexanediol), and the last letter, the type of VOC (L—limonene, P—pinene, and T—toluene). Letters in between indicate the type of isocyanate used in the formulation (I - IPDI and T stands for TDI and Tubassist). The presence of catalysis DBTL in the sample is not indicated in the abbreviation.

In the following, the names of the samples are given as abbreviations in the text. The letters correspond to the composition of the sample. The first letter stands for the type of hydrophilic monomer (G, glycerin; HD, hexanediamine; and H, hexanediol), and the last letter stands for the type of VOC (L—limonene, P—pinene, and T—toluene). The letters in the middle of the abbreviation indicate the type of isocyanate used in the formulation (I stands for IPDI and T stands for TDI and Tubassist). The presence of catalytic DBTL in the sample is not indicated in the abbreviation.

### 2.3 Characterization of Capsules

The average size of O/W droplets was analyzed by an optical microscope (Olympus BX60). The average size and the size distribution of air-dried capsules were determined from scanning electron microscopy (SEM) images using ImageJ for analysis and retracing the shape of capsules. All SEM images of synthesized capsules were acquired on an LEO (Zeiss) 1550 VP at an accelerating voltage of 5 kV. A drop of the diluted sample was deposited on a silicon wafer and dried at room temperature. To enhance the electron density contrast, samples were coated with a thin layer of gold before measurement.

The chemical composition and thermal properties of the capsules were studied by Fourier transform infrared (FTIR) spectroscopy and thermogravimetric analysis (TGA). For the FTIR and TGA measurements, the capsule samples were dried under ambient conditions for 24 h (day 1 sample) and then kept in a thermal cardboard with circulated air at 50 °C for 15 and 30 days (samples, day 15 and day 30). FTIR spectra were recorded from 400–4000 cm^–1^ at room temperature using attenuated total reflection (ATR) method with a Bruker Alpha-P instrument. Analyses were carried out by placing dry capsule samples (2–4 mg) directly into the ATR head.

The thermal stability analysis and evaluation of VOC content were done using a thermogravimetric analyzer (TGA, Exstar TG/DTA7000) over the temperature range of 25–800 °C under a nitrogen atmosphere and at a heating rate of 10 °C/min.

### 2.4 Emission Testing

The emission testing was carried out in emission test chambers according to EN 16516.^11^ About 1 mL of microcapsule suspension was pipetted into a Petri dish. The Petri dish was then placed into a glass desiccator (i.e., the emission test chamber) with a volume of 24 L. The chambers were operated dynamically under controlled climatic conditions at a temperature of 23 ± 1 °C, a relative humidity of 50 ± 5%, and an air exchange rate of 0.5 per h. The test chambers were equipped with a ventilator to ensure homogeneous air distribution. All chambers provided sampling ports for pumped sampling into the thermal desorption tubes. Before being loaded, the chambers were tested for blank values.

Air sampling started 3–4 days after loading, and the emission testing usually lasted 2–3 weeks. Sampling was conducted every second day with the help of a sampling pump, a mass flow meter, and Tenax TA thermal desorption tubes. About 1 L of test atmosphere was sampled (sampling flow rate of 100 mL/min).

### 2.5 Thermal Desorption-Gas Chromatography-Flame Ionization Detection Analysis (TD-GC-FID)

The loaded Tenax TA tubes (Gerstel, Mülheim/Ruhr, Germany) were analyzed by a GC (6890N, Agilent) coupled to a flame ionization detector (FID) and equipped with a thermal desorber (TD, TDSA 2, Gerstel) with a two-stage desorption. In the first stage, the sorbent tube was heated, and the desorbed VOC components were transferred and concentrated into a cold injection system (CIS, CIS 3, Gerstel) at −100 °C. The temperature program used for the sorbent tube desorption started at 40 °C (hold 1 min) and was heated with a rate of 40 °C/min to 280 °C (hold 5 min). In the second stage, after the collection of the desorbed substances from the TD oven was finished, the cold trap was quickly heated at a rate of 12 °C/s to 280 °C (hold 3 min) so the compounds were released and transferred to the GC column. The GC column was an RTX-Volatiles (60 m length, 0.32 mm inner diameter, 1.5 μm film thickness, Restek). A constant pressure of 1.3 bar (equal to about 2.5 mL/min flow) and He (99.9999%, Linde) as a carrier gas were used. The effluent was introduced (splitless) into the GC column, and the temperature program of the GC started at 40 °C (hold 5 min), was then heated with a rate of 4 °C/min to 80 °C, then heated with a rate of 7 °C/min to 180 °C, and then heated with a rate of 10 °C/min to 260 °C (hold 4 min). The FID was operated at a temperature of 280 °C, with a flow of 35 mL/min H_2_, 350 mL/min synthetic air, and 42.5 mL/min He makeup gas.

The TD-GC-FID system was calibrated at the beginning and end of each month. In detail, the external calibration was performed by preparing a solution of different VOCs (toluene, d-limonene, and α-pinene, among others) in methanol. The solution was diluted to yield five concentrations (20, 100, 180, 260, 340, and 420 ng/μL). One μL of each calibration standard was then spiked into individual Tenax TA tubes, which were measured by the TD-GC-FID. Additionally, the second calibration standard (100 ng) was measured twice a week as a QC standard.

### 2.6 Evaluation of Emission Testing Results

For a comparison of the measurement results between different experiments and an evaluation of the impact of the progress relevant parameters, the mass-specific emission rate (SER_*m*_) was used, as this is a normalized parameter.

The SER_*m*_ in μg/g h is calculated according to eq 1, where *c*_*i*_ is the test chamber air concentration of VOC *i* in μg per m^3^, *n* is the air exchange rate per h, *L* is the chamber loading in g per m^3^,  is the air flow through the chamber in m^3^ per h, *m* is the mass of the impregnated material loaded into the chamber, and *V*_ch_ is the test chamber volume in m^3^.
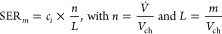
1

## 3 Results and Discussion

### 3.1 Synthesis of Microcapsules

In this study, three VOCs (limonene, toluene, and pinene) were used as a “core material” for encapsulation. The first series of experiments was performed with the SPG membrane having an average pore size of 5.1 μm. According to the manufacturer’s information, this type of membrane forms droplets with an average size of 15 μm. In the second set of experiments, the SPG membrane with a pore size of 9.9 μm was applied for some preselected limonene-based formulations to form capsules above 50 μm in diameter. Three different isocyanate-containing monomers (IPDI, TDI, and TUBASSISTFIX 157W) and three different hydrophilic monomers (glycerin, 1,6-hexanediol, and 1,6-HD) were used in different ratios to find the optimal formulation for colloidally stable VOC-loaded capsules with homogeneous size distribution. It became clear after preliminary tests that the synthesis parameters had to be optimized for each VOC because of their specific properties (vapor pressure, miscibility with water and isocyanate(s), and interfacial VOC/water tension). In a general procedure, the miscibility of the components in the organic phase was first evaluated. A stable emulsion was then prepared from both phases while examining the surfactant effectiveness. Finally, the interfacial polyaddition–polycondensation reaction was carried out. In detail, Tubassist is completely miscible with toluene; however, it is soluble only up to 12 and 15 vol % in limonene and pinene, respectively. All other organic components were miscible with each other. With respect to the emulsions, a highly concentrated O/W ratio of about 1:7 was considered first to prevent the coalescence of the droplets and the aggregation of capsules during the polymerization process. However, the formulation recipe had to be slightly modified for the synthesis of large capsules, using a 9.9 μm membrane. The amount of aqueous phase was increased up to an O/W ratio of about 1:27, and an additional anionic surfactant, SDS, was used to provide effective stabilization for the large size droplets. H. Yoshizawa et al.^34^ successfully applied this system previously for the preparation of cross-linked polymer microspheres through suspension copolymerization of polyethylene glycol monomethacrylate and ethylene glycol dimethacrylate using the SPG membrane of 9 μm.

**Figure 2:**
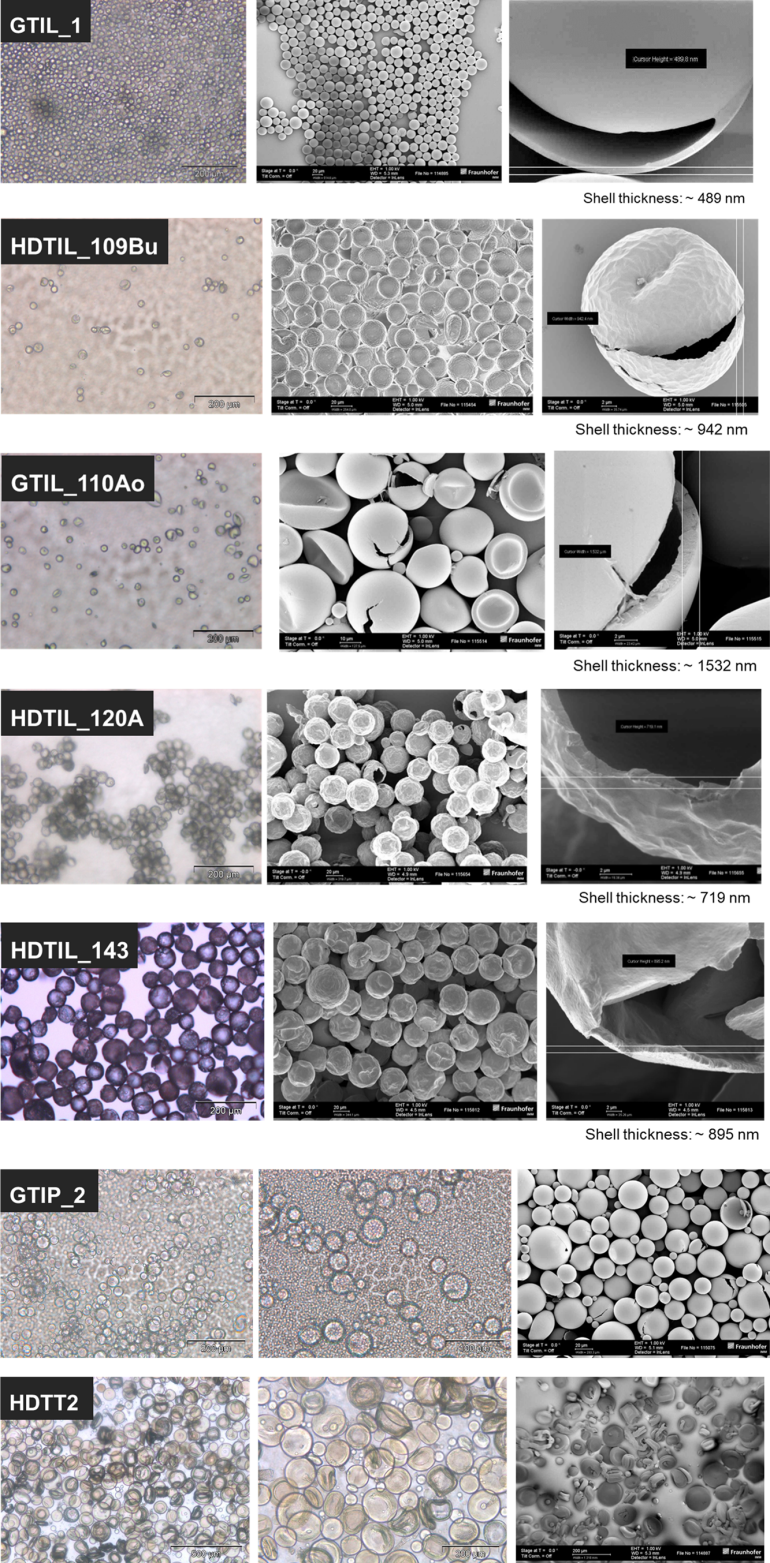
Light microscopy and SEM images of different VOC-loaded polymer capsules.

The first assessment of successful emulsification and encapsulation was carried out visually. The samples were further characterized by optical and electron microscopy, in which no phase separation was observed during emulsification, and no layer of free VOC was visible at the end of the polymerization. The samples with more than 10% broken capsules (based on the total number of capsules observed during microscopy examination) were excluded for further characterization. The chemical composition and average size of successfully produced, colloidally stable capsules are summarized in Table 1. The optical and SEM images of the selected samples are shown in Figure 2.

As a result, with a 5.1 μm SPG membrane, stable and size-homogeneous capsules were generated with all VOCs only with the amounts of ingredients listed in Table 1. Limonene- and pinene-loaded capsules with diameters below 20 μm were prepared using IPDI/Tub and glycerin as cross-linkers and hydrophilic monomers, respectively. Furthermore, the use of HD and hexanediol (but only for pinene) also leads to capsules with an average diameter of 23 μm. In the system of limonene and hexanediol, capsules with very thin shell were obtained, which were not mechanically stable. Increasing the amount of cross-linker/hydrophilic monomer also did not result in stable capsules.

The same composition of organic phase was not working to prepare toluene-loaded capsules; therefore, an additional catalyst DBTL was added (samples HTIT_1 and GTIT_1). Furthermore, the encapsulation of toluene using HD failed when IPDI/Tub (with or without DBTL) and PVA as surfactant were used; a complete coagulation was observed. Therefore, we used only the oligomeric diisocyanate Tubassist as a cross-linker and replaced the nonionic surfactant with anionic SDS to increase the stability of the droplets/capsules (sample HDTT_2). All obtained toluene-containing capsules are larger compared to the limonene- and pinene-containing ones. One of the explanations could be related to the high vapor pressure of toluene (29 hPa at 20 °C) compared to those of limonene (2 hPa at 20 °C) and pinene (5 hPa at 20 °C), which leads to coalescence of the droplets. Another explanation could be the swelling of the polyurethane/polyurea shell due to the low solvent–polymer interaction parameter of 0.33–0.48, which was found for benzene, for example.^35^ To investigate the effect of shell thickness on the VOC emission profile, two types of limonene-loaded capsules with a reduced amount of organic phase were synthesized (samples GTIL_110Ao and HDTIL_109Bu). The average size of the capsules obtained with HD was in the range of 24 μm, while the capsules obtained with glycerin had an average diameter of about 43 μm. Although the stability of the droplets should not be affected by the reduced O/W ratio, it looks as if the droplets have been fused together.

When the SPG membrane with a pore size of 9.9 μm was used, intact and stable capsules could only be produced with HD. An explanation could be the higher reactivity of the isocyanate and amine groups compared to the hydroxyl groups of hexanediol and glycerin. The reactivity of nucleophiles in reactions with diisocyanates is influenced by several factors, including the type of nucleophile.^36^ Amines are strong nucleophiles and react very rapidly with diisocyanates, forming ureas. Alcohols are less nucleophilic than amines but are still quite reactive with diisocyanates, leading to the formation of carbamates (urethane linkages).

To further reduce the coalescence of droplets, the reaction temperature was reduced from 60 to 45 °C, and the catalyst DBTL was added to the formulation with IPDI (sample HDTIL_120A). In two other formulations (HDTTL_133A and _143), TDI was used as a cross-linker instead of IPDI to increase the polymerization rate and decrease the reaction time. The reactivity of the isocyanate groups in TDI is higher than that in IPDI, due to the electron-withdrawing effect of the aromatic ring.^37^

The “core–shell” morphology could be seen in all samples analyzed by microscopy (Figure 2). Both intact and burst particles were observed after exposure to the high-vacuum conditions required for SEM imaging, confirming their hollow nature. Furthermore, limonene-loaded capsules with different shell thicknesses were obtained by adjusting the reaction composition. The capsule’s surface synthesized with HD has a higher roughness than capsules produced with glycerin or hexanediol. The microscopic images also demonstrate that the shell of the capsules containing toluene is deformed, presumably soft, which could be attributed to the effect of toluene as a plasticizer.

### 3.2 Chemical Structure of the Microcapsules

**Figure 3:**
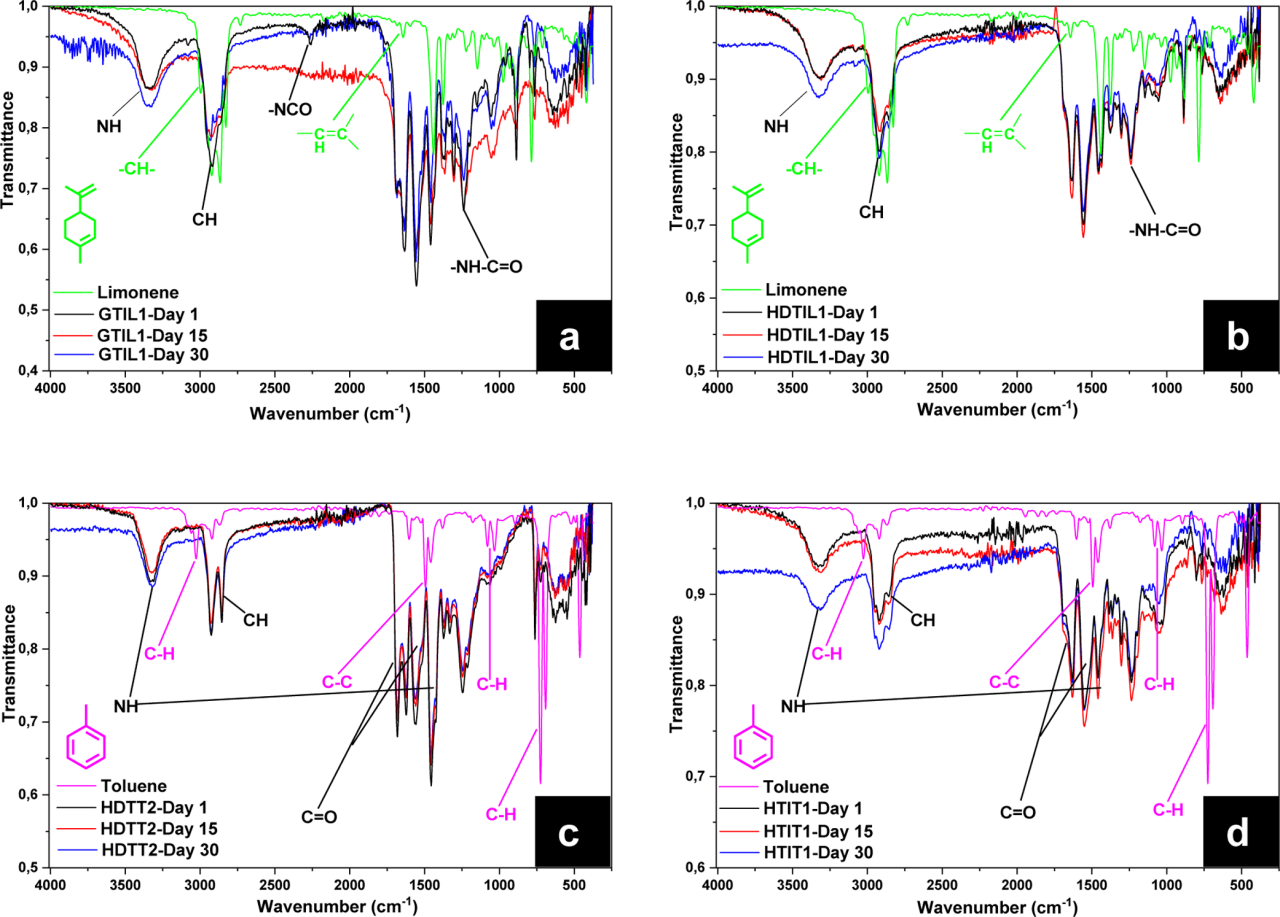
FTIR spectra of limonene-loaded capsules GTIL_1 (a) and HDTIL_1 (b) and toluene-loaded capsules HDTT_2 (c) and HTIT_1 (d) after drying at 50 °C for different time periods.

The chemical structure of the capsules was confirmed by FTIR spectra. As an example, the FTIR spectra are presented of two different limonene- (GTIL_1 and HDTIL_1) and toluene- (HDTT_2 and HTIT_1)-loaded capsules measured after drying for different time periods (Figure 3).

The presence of functional groups that belong to polyurethane, polyurea, and limonene or toluene can be clearly observed. The IR spectra of all capsules exhibit characteristic peaks at 2900 (C–H stretching and –CH_3_ group), 1649 (C–C multiple bond stretching and –C–CH_2_ group), 1432 (C–H bonding and –CH_3_ group), and 1400 cm^–1^ (C–H bonding and –CH_3_ group). The broad peak at 3345 cm^–1^ is associated with the stretching vibration of N–H bonds in the urethane and urea groups,^38,39^ which constitute the polymer capsule, while the peaks at 1692–1643 cm^–1^ correspond to typical carbonyl absorption peaks of ester bonds. The peaks at 2900 cm^–1^ are attributed to the stretching vibrations of alkane –CH groups, which form another characteristic group of polyurethane.^40,41^ Although no HD was present in the formulations of the GTIL_1 and HTIT_1 samples, the characteristic peaks belonging to both urethane and urea groups are present in the spectra. This is due to the fact that water is a weak nucleophile and also reacts with diisocyanates, forming urea and carbon dioxide.^42^ The presence of non-reacted isocyanate groups could be seen on FTIR spectra of the GTIL_1 sample after 1 day of drying, but after 15 days, all groups were reacted, indicating completed polymerization of the shell.

Additionally, due to the overlap of the signals of limonene^43^ and toluene^44^ with those of the polymer capsules, it was not possible to determine their evaporation by FTIR measurements alone. However, from the FTIR results measured after three different time intervals, it can be concluded that no structural degradation occurred with the capsules within these time periods.

### 3.3 Thermal Stability of Microcapsules

**Figure 4:**
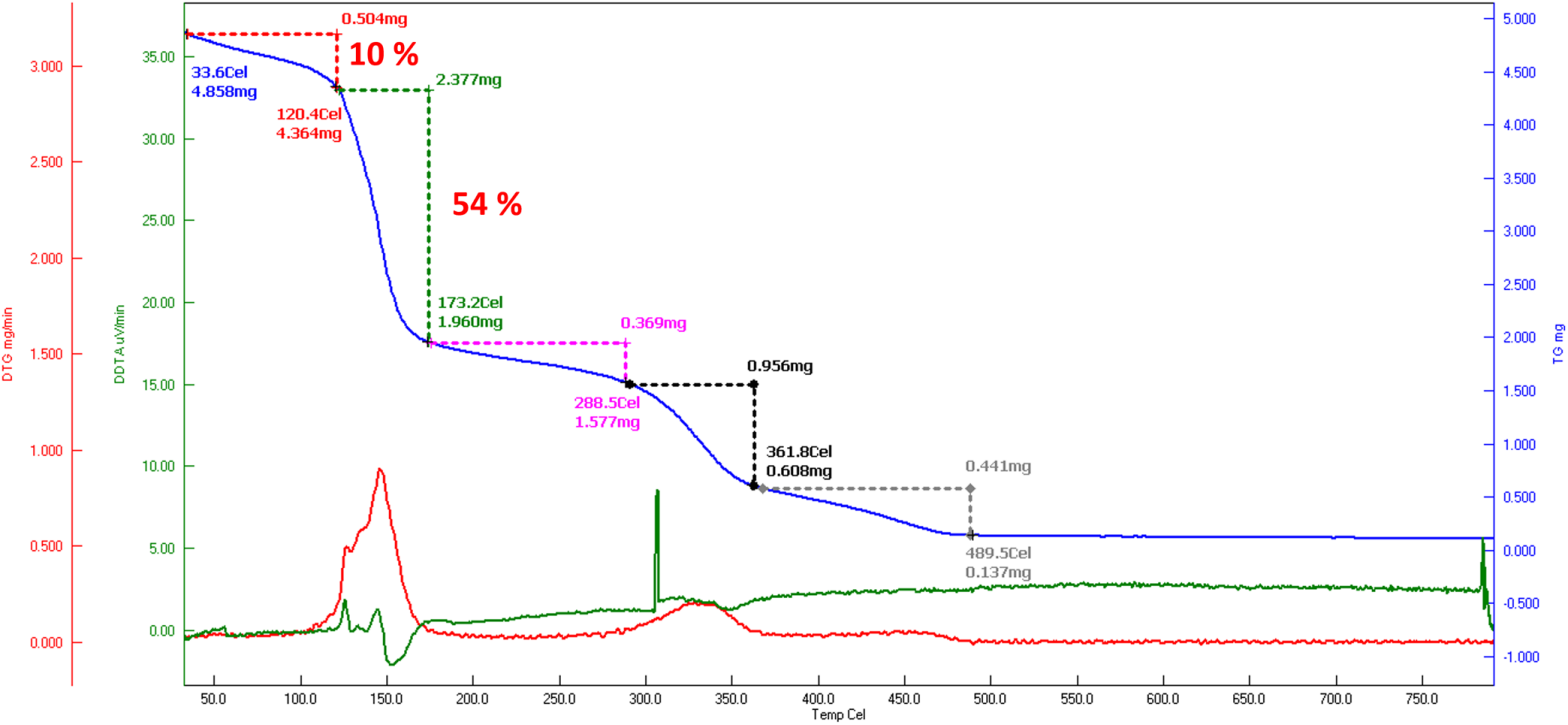
TGA results of limonene-loaded microcapsules GTIL_1 after 1 day of drying.

Capsules with limonene and toluene were further characterized by TGA. Polyurethane typically exhibits a two-stage degradation process. In the first stage (200–350 °C), the decomposition of hard segments (urethane groups), involving depolymerization and breaking of urethane bonds, takes place. In the second stage (350–500 °C), the decomposition of the soft segments (polyol backbone) typically leading to further weight loss as the remaining polymeric components break down.^45^ Polyurea follows a similar but slightly higher temperature range due to the stronger urea linkages: at 250–350 °C, degradation of urea bonds takes place, and in the temperature range 350–550 °C, decomposition of the remaining soft segments and further chain scission is observed. Figure 4 shows the TGA curve (blue line) of the GTIL_1 microcapsules. It reveals five distinct breakpoints of mass loss. The first region, denoted by red dots, spans from 30 to 120 °C and exhibits a mass loss of ∼10%, which is attributed to the presence of water.^46,47^ The second region, indicated by green dots, displays a significant mass loss of about 54% at 120–173 °C, which corresponds to limonene evaporation.^26,47^ The initial decomposition temperature of the polymer shell was around 200 °C, and it was completely decomposed at 500 °C.^47,48^ Several stages of polymer decomposition that are attributed mainly to polyurethane could be seen. The residual residue is below 5%.

**Figure 5:**
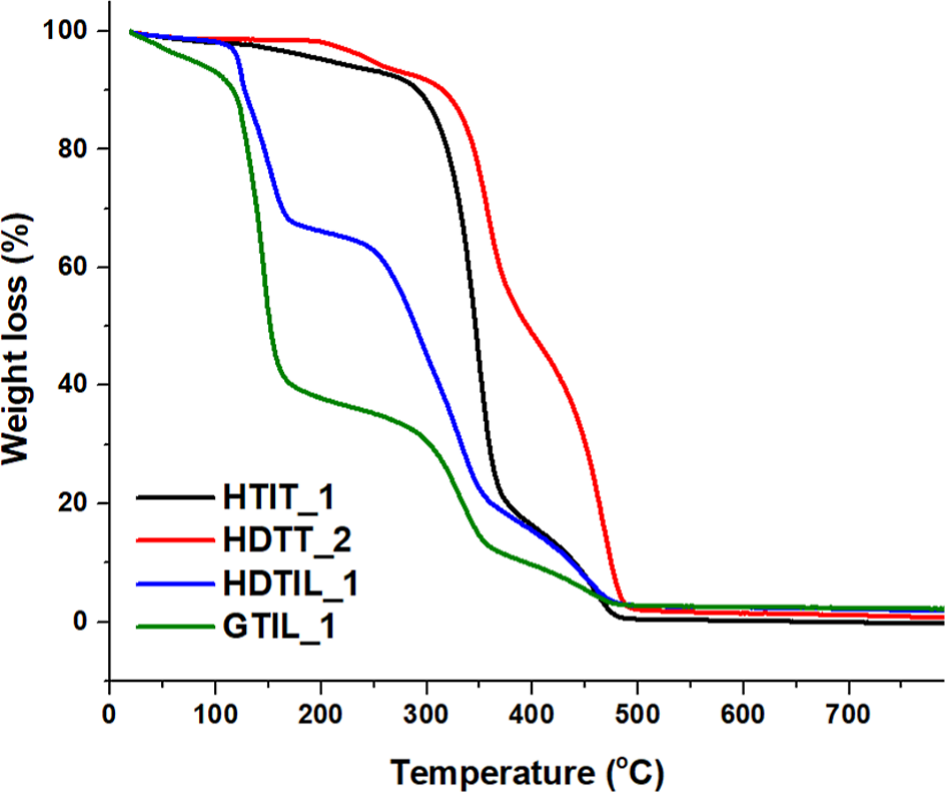
TGA curves of different limonene- and toluene-loaded microcapsules dried for 1 day.

A similar mass loss trend was observed for all samples loaded with limonene. Specifically, the amount of evaporated limonene detected on day 1 was between 18 and 54%. In contrast, the percentage of toluene was about 7–9% in all capsules examined. The TGA curves of the most representative limonene- and toluene-loaded microcapsules, measured on day 1, are shown in Figure 5. It could be seen that the toluene-containing capsules degraded at temperatures higher than those of the limonene-containing ones. The degradation temperature of the capsules prepared in the presence of HD is also higher, indicating the presence of more urea bonds in the shell and a higher cross-linking density, as the tightly bound network structure delays degradation.^45^ In fact, the sample HDTT_2 was prepared in the presence of oligo-isocyanate Tubassist, so a higher cross-linking of the shell can be expected.

**Figure 6:**
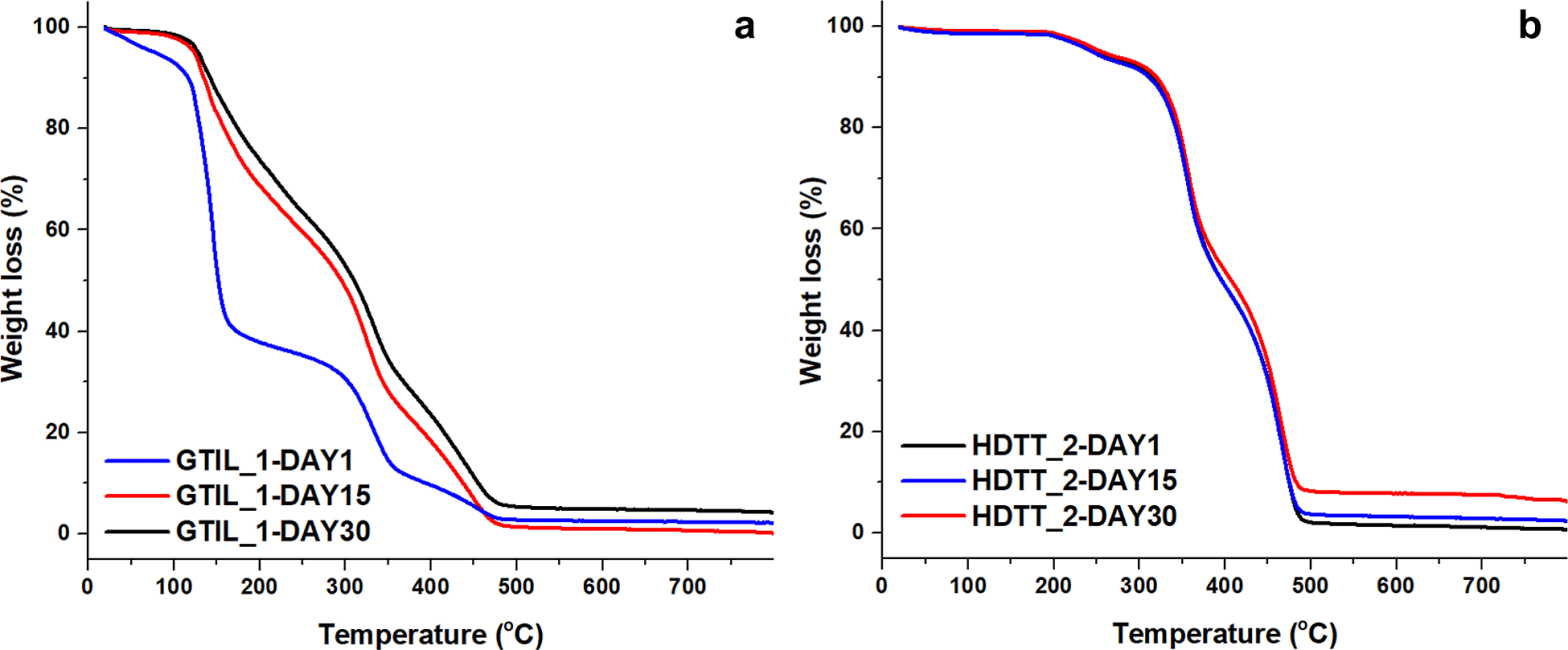
TGA curves of samples GTIL_1 (a) and HDTT_2 (b) after different drying times.

The permeability of the polymeric shell could not be determined by BET because the required stable vacuum could not be obtained due to persistent emission of VOC from the capsule. Therefore, the capsule samples were subjected to long-term drying (up to 30 days) at a constant temperature of 50 °C, and TGA was performed at different time periods. As a result, the amount of limonene decreases by 50–55% on day 1. The mass loss was less dominant after 15 days of drying; however, it exhibits a stable behavior afterward since the curve is similar after 30 days of drying (Figure 6a). In the case of toluene-loaded capsules, almost no difference was observed between days 1 and 30 (Figure 6b). The data obtained indicate that the shell of the microcapsules loaded with limonene contains pores and that limonene can be released from the microcapsules. Furthermore, despite the absence of any significant changes in the FTIR signals of the capsules over the designated time intervals, the mass loss observed in the TGA indicates that the limonene compound released over time. At the same time, the shell of the toluene-loaded capsule type is more sealed.

### 3.4 Emission Testing

**Figure 7:**
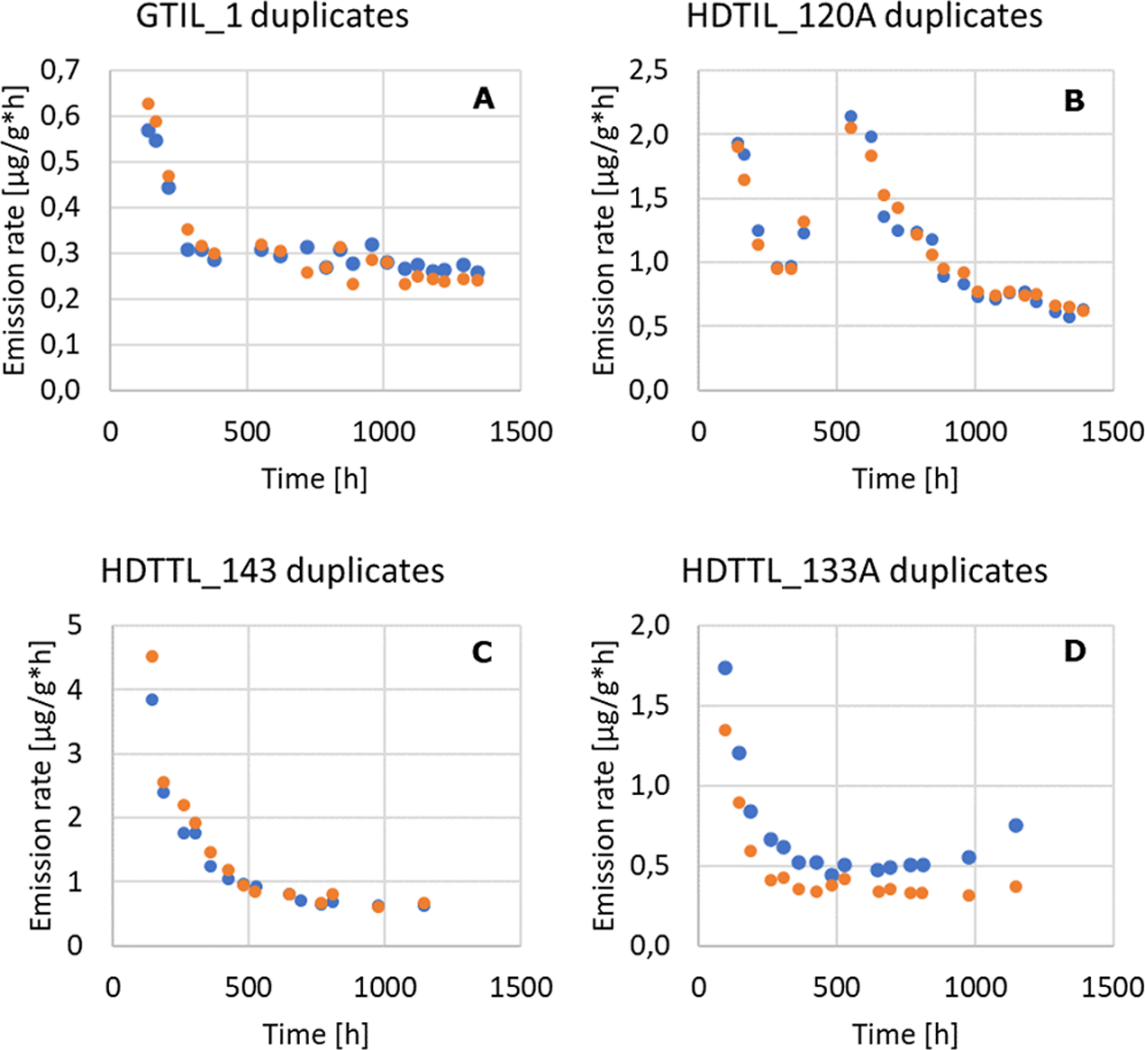
Two replicate testings of different limonene-loaded capsules GTIL_1 (A), HDTIL_120A (B), HDTTL_143 (C), and HDTTL_133A (D).

The microcapsules developed herein are to be used as ERMs. Two goals were defined: to reach a suitable/well detectable concentration in the test chamber and a stable emission of less than 10% deviation over 14 days. The concentration can be easily adjusted by increasing the sample mass, so the main goal was to optimize the time-dependent release of VOC from the capsules. Most building materials exhibit a decaying emission curve, and most tested microcapsules revealed the same behavior. The results from the emission testing are summarized in Table 2 and shown in Figure 7.

**Table 2:** Microcapsule Emission Change over 14 Days and Average Emission Rate (ER)

sample name	ΔER, %	mean ER, ng/g h	VOC
GTIL_1	89 (decrease)	280	limonene
GTIL_1^a^	5 ± 3 (decrease)	273 ± 1	
HDTIL_1	70 (increase)	170	
GTIL_110Ao	87 (decrease)	2610	
HDTIL_109Bu	100 (decrease)	171	
HDTIL_120A	279 (increase)	1580	
HDTIL_120A^a^	13 ± 1 (decrease)	800 ± 30	
HDTTL_133A^a^	29 ± 23 (increase/decrease)	450 ± 100	
HDTTL_143^a^	10 ± 1 (decrease)	708 ± 18	
HTIT_1	73 (decrease)	200	toluene
GTIT_1	57 (decrease)	82	
HDTT_2	48 (decrease)	7	
GTIP_2	56 (decrease)	45	pinene
HDTIP_1	44 (decrease)	76	
HTIP_1	63 (decrease)	26	

a Long-term and duplicate emission testing.

A change of 48–73% over 14 days was found for the toluene-containing samples with an average emission rate of 7–200 ng/g h. A deviation of 44–63% over 14 days was found for the pinene-containing samples with an average emission rate of 26–76 ng/g h. In the case of limonene-loaded capsules, the samples HDTIL_1 and GTIL_1 exhibited the same initial behavior, but the emission curve of HDTIL_1 showed a local minimum at ∼160–190 h, followed by an increase of emission (similar to Figure 7B). It is important that the suspension is well mixed during pipetting so that reproducible results are obtained, because broken and intact capsules rapidly separate by floating and setting, respectively.

To further stabilize the emission, limonene-loaded capsules with a thicker shell were synthesized. However, it was found that the emission change over 14 days does not differ much between capsules with different shell thicknesses (sample GTIL_1 and GTIL_110Ao with shell thicknesses of 489 and 1532 nm, respectively). Moreover, the average emission for GTIL_110Ao was much higher than that for GTIL_1 (2610 vs 280 ng/g h). It seems that the porosity and diffusion of VOCs are not directly related to the shell thickness. It could be that the size of the capsules had an effect on the emission. Considering all capsules loaded with limonene, there is a tendency for larger capsules (43–60 μm) to have a higher emission rate [450–2610 ng/(g h)] than smaller capsules (16–24 μm, 170–280 ng/g h). This trend is independent of the chemical composition of the capsule and the shell thickness and is therefore ascribed to the larger limonene reservoir of the larger capsules. In general, the measured stabilities for limonene emission over 14 days were between 70 and 279%.

For practical reasons, most of the emission tests were conducted for 2–3 weeks. However, the testing duration can have a significant influence on the performance of a material, especially for materials that have not yet fully reached their stable phase.^49^ Therefore, four samples were tested for a prolonged period (up to 60 days), and only the stable phases were compared to diminish doubts about incomplete equilibrium states and starting points. Both replicate testings (parallel testing in separate chambers) of GTIL_1, HDTIL_120A, HDTTL_143, and HDTTL_133A are plotted in Figure 7.

Most replicate measurements show only a small deviation from each other. The most stable result was obtained for GTIL_1 (5% change in emission over 14 days with a mean emission of 270 ng/g h; Figure 7A). The samples produced with HD, HDTIL_1 and HDTIL_120A, exhibit unique emission behavior. Initially, the emission decreases but then increases. This behavior is not dependent on the size of capsules (i.e., 21 ± 10 nm vs 58 ± 6 nm). After a second local maximum (∼550 h), the emission of HDTIL_120A starts to decrease again, reaching a more stable phase than before (1000–1400 h; 14% decrease of emission over 14 days).

The most reproducible result with good stability was obtained by testing HDTTL_143 (10% change in emission over 14 days with a mean emission rate of 620 ng/g h). Including the obtrusive last two data points, the capsules HDTTL_133A exhibit the lowest reproducibility—the values were 6% decrease and 53% increase, respectively, over 14 days (Figure 7D).

Overall, two limonene-loaded capsule samples, GTIL_1 and HDTTL_143, fulfill the requirement of ≤10% change over 14 days. Additionally, sample HDTTL_143 showed a high reproducibility and thus was further used for another reproducibility and stability study with prior pre-aging (storage in dry air for 14 days).^50^ It was proven that HDTTL_143 microcapsules do not change over time, at least in their pre-aged form, and have a great potential to be used as an ERM.

## 4 Conclusions

In this study, poly(urethane–urea) microcapsules loaded with various VOCs were synthesized by interfacial polymerization using the SPG membrane technology. Depending on the VOC type, the synthesis parameters had to be adjusted individually in order to obtain colloidally stable capsules with the required properties (particle size, encapsulation efficiency above 80%, low polydispersity). The emission tests were carried out on capsules with different surface morphologies and chemical compositions. The type of VOC, the capsule size/composition, and the shell thickness: all these factors have an influence on the emission. In order to formulate the VOC-loaded polyurethane/polyurea capsules, it is important to use the proper surfactant that prevents the droplets coalescence, the VOC should be hydrophobic enough to reduce the Ostwald ripening, and the VOC should not have any groups that can react with isocyanate groups. Furthermore, it is important that VOC is miscible with the organic phase and does not dissolve/swell the polymeric shell. The targeted stable emission (ΔER ≤ 10% over 14 days) was achieved with two capsule samples (GTIL_1 and HDTIL_143) loaded with limonene as VOC. Capsule sample HDTIL_143 is stable for up to 12 months when pre-aged and stored in airtight packaging. However, to achieve the stable emission phase from the capsules, they need to be pre-aged, the time that is required to remove any VOC from the shell, open all available pores, and equilibrate the diffusion rate. Future improvements might concern the aging (e.g., by improving the pre-aging) and the scope of additional VOCs.

## Abbreviations

ATR: attenuated total reflection
DBTL: dibutyltin dilaurate
EN 16516: European Standard 16516
ER: emission rate
ERM: emission reference material
FTIR: Fourier-transform infrared spectroscopy
ISO: International Organization for Standardization
IPDI: isophorone diisocyanate
O/W: oil-in-water
PTFE: polytetrafluoroethylene
PVA: poly(vinyl alcohol)
QA/QC: quality assurance/quality control
SDS: sodium dodecyl sulfate
SEM: scanning electron microscopy
SPG: Shirasu porous glass
TDI: tolylene 2,4-diisocyanate
TGA: thermogravimetric analysis
VOC: volatile organic compound
